# Thymus research in relation to myasthenia gravis: a new perspective on cell subpopulations and future directions

**DOI:** 10.3389/fimmu.2025.1649171

**Published:** 2025-10-14

**Authors:** Naoko Matsui, Izumi Ohigashi, Alexander Marx

**Affiliations:** ^1^ Department of Neurology, Tokushima University Graduate School of Biomedical Sciences, Tokushima, Japan; ^2^ Division of Experimental Immunology, Institute of Advanced Medical Sciences, Tokushima University, Tokushima, Japan; ^3^ Laboratory of Developmental Immunology, Institute of Photonics and Human Health Frontier, Tokushima University, Tokushima, Japan; ^4^ Institute of Pathology, University of Gottingen, Gottingen, Germany

**Keywords:** myasthenia gravis, thymectomy, thymic epithelial cell, thymic follicular hyperplasia, thymoma

## Abstract

The thymus generates T cells from immature thymocytes and prevents autoimmune diseases through negative selection and the generation of FOXP3^+^ regulatory T cells (Tregs). The thymic architecture is typically divided into two distinct microenvironments, the cortex and the medulla. These microenvironments are characterized by the presence of cortical thymic epithelial cells (cTECs) and medullary thymic epithelial cells (mTECs), respectively. Recent single-cell and spatial transcriptomic analyses have revealed the expanding diversity of TEC subpopulations in mice and humans. Myasthenia gravis (MG) is an autoimmune disorder characterized by fatigue resulting from muscle weakness, which is caused by antibodies toward structures within the neuromuscular junction. The most common target of pathogenic autoantibodies in MG is the acetylcholine receptor (AChR). MG patients are prone to thymic abnormalities, including thymic follicular hyperplasia and thymoma. Previous studies have suggested that mTECs expressing major histocompatibility complex (MHC)/AChR–peptide complexes are involved in the intrathymic pathogenesis of this MG type. However, the exact mechanisms are unknown. This review provides an update on the diversity of TEC subpopulations and other cellular alterations in the MG thymus. Additionally, we present hypotheses on the pathogenetic pathways leading to MG and suggest potential future directions in thymus research.

## Introduction

The fortuitous association between the thymus and myasthenia gravis (MG) was first reported in 1939, when the excision of a cystic thymic tumor had a strikingly positive impact on myasthenic symptoms in a patient with MG (1). A study performed in 1949 indicated that thymic abnormalities, including hyperplasia and thymoma, were detectable in approximately 90% of patients with MG, and that thymectomy for non-thymoma was effective in 43 to 50% of patients (2). Since then, thymectomy has remained a crucial therapeutic intervention for MG patients with thymic abnormalities and an oncological necessity for patients with thymomas (3, 4), despite the emergence of new and highly effective therapeutic agents in recent years (5).

Notwithstanding the discovery of a link between thymus pathology and MG in 1949, as mentioned above, the belief persisted that the thymus was actually a superfluous organ, as its involution from childhood onwards seemed to have no consequences—until its crucial role in T cell production was recognized in 1961 (6). The function of the thymus largely depends on the function of thymic epithelial cells (TECs). Over the past two decades, our understanding of the mechanisms regulating TEC function and development has significantly advanced. Single-cell and spatial transcriptomic analyses have revealed a far greater diversity of TEC subpopulations than previously recognized (7, 8). However, most of these studies have been conducted using mouse thymuses. Therefore, comprehending the similarities and differences between mouse and human thymuses is crucial for clinicians seeking to understand human diseases associated with thymic abnormalities.

In this review, we summarize current advances in our understanding of TEC properties in mouse and human thymuses, as well as the pathomechanisms of MG thymus. We also discuss future directions in thymus research, including potential new therapeutic strategies and the development of appropriate biomarkers for MG.

## Thymic epithelial cells in mouse and human thymuses

The thymus consists of two microenvironments, the cortex and the medulla, whose functions are characterized by the roles played by cortical TECs (cTECs) and medullary TECs (mTECs), respectively. cTECs express functional molecules, including IL7 and Dll4, that regulate early T cell development. Furthermore, cTECs express enzymes that produce self-peptide antigens, inducing the positive selection of T cells. These enzymes comprise the β5t-containing thymoproteasome, the thymus-specific serine protease, and cathepsin L (9, 10). On the other hand, mTECs regulate the establishment of T cell self-tolerance. For example, chemokine CCL21 produced by mTECs is involved in the migration of positively selected cortical thymocytes into the medulla, whereas the nuclear factor AIRE regulates the expression of tissue-specific antigens for the negative selection of self-reactive T cells and the generation of regulatory T cells (Tregs) (11–13). The importance of these cTEC- and mTEC-associated molecules in thymic function has been revealed through the analyses of animal models, particularly genetically modified mice. However, transcriptomic analysis of human TECs has demonstrated that cTEC- and mTEC-associated molecules are similarly present in human cTECs and mTECs, respectively (14, 15), indicating that similar molecular mechanisms govern the regulation of TEC functions in humans and animal models. These analyses have also revealed differences between human and mouse TECs, such as IL-25 expression, which characterizes mouse but not human tuft cells—a subset of mTECs present in both species.

### Thymic epithelial progenitors in mouse and human thymuses

During embryonic thymus organogenesis, TEC emergence begins on embryonic day 11 in mice and the sixth week of gestation in humans, marked by the expression of Foxn1, a landmark transcription factor (16). Intrathymic transplantation of single TECs isolated from fetal mouse thymus, along with lineage tracing of transplanted TECs, has revealed that cTECs and mTECs are derived from a common TEC progenitor (17, 18). Bipotent TEC progenitors have also been identified in the adult mouse thymus (19, 20). In addition to bipotent TEC progenitors, mTEC-specific progenitors, including RANK^+^ TECs, Krt19^+^ TECs, and Sox9^+^ TECs, as well as Cldn3,4^high^SSEA1^+^ mTEC stem cells with self-renewing and clonogenic potential, have been unveiled in the fetal mouse thymus (21–25). Recently, we reported that mTECs expressing CCL21 in the fetal mouse thymus are capable of giving rise to AIRE^+^ mTECs (26), suggesting that the functional conversion of thymocyte-attracting mTECs into self-antigen-presenting mTECs contributes to the establishment of a functional medullary microenvironment.

In humans, TEC stem cells have been identified in the postnatal thymus (27, 28) through single-cell RNA sequencing of cTECs and mTECs, within a TEC cluster termed Polykeratin (PolyKRT). PolyKRT expresses multiple cytokeratins, including KRT5, KRT8, KRT13, KRT14, KRT15, KRT17, KRT18, and KRT19. The PolyKRT cluster is predominantly localized in the subcapsular and perivascular regions of the thymus. It exhibits long-term expansion potential *in vitro*, as well as the capacity to differentiate into multiple TEC lineages (28). However, it remains unclear whether the frequency and differentiation capacity of PolyKRT cells change with aging or in the context of thymic abnormalities. In addition, it is unknown whether lineage-restricted progenitor populations, such as Krt19^+^, RANK^+^, and CCL21^+^ embryonic TECs identified in the mouse thymus, are also present in the human thymus. Further studies are needed to elucidate the roles of human TEC stem and progenitor cells in thymic involution and the pathogenesis of thymus-associated diseases.

### TEC subpopulations other than progenitors in mouse and human thymuses

As far as the mouse thymus is concerned, it is well known that mTECs are a heterogeneous population that is roughly divided into two subpopulations: mTEC^low^ (CD80^low^ MHC II^low^), which includes immature mTECs and those in the post-AIRE stage, and mTEC^high^ (CD80^high^ MHC II^high^), a mature mTEC subset that includes AIRE^+^ mTECs. The heterogeneity of cTECs has also been recognized on the basis of the expression of cTEC-associated molecules, such as CXCL12 and DLL4 (29, 30). However, single-cell transcriptomic analyses of TECs isolated from the mouse thymus have revealed a far greater diversity of mTEC subsets, including thymic mimetic cells, which exhibit transcriptional and epigenetic signatures resembling those of extrathymic cells and in the post-AIRE stage (7, 8, 31–33). These thymic mimetic cells are suggested to contribute to T cell tolerance by presenting self-antigens that are typically expressed in peripheral tissues (8). The functions of thymic mimetic cells beyond antigen presentation have also been reported, including the regulation of invariant NKT2 cell development and function by thymic tuft cells, the control of thymic cellularity by endocrine mTECs, and the generation of IgA^+^ plasma cells in the thymus by microfold mTECs (31, 33, 34).

Similar to mouse TECs, the diversity of human TEC subpopulations has been confirmed by single-cell transcriptomic analyses. These include various mTEC subpopulations, such as CCL21^+^ mTEC^low^/mTEC-I, AIRE^+^ mTEC^high^/mTEC-II, and mimetic TECs, as well as a limited number of cTEC subpopulations (14, 15, 35). Immature TECs, which are committed to neither the cTEC nor the mTEC lineage, have also been detected in the human thymus (15, 35). These cells express KRT15, which is also found in PolyKRT human TEC stem cells (28). Spatial mapping of TECs has indicated that immature TECs are located in the subcapsular area of the fetal thymus and in both the subcapsular area and the cortico-medullary junction of the pediatric thymus (35). The shift in immature TEC localization from fetal to pediatric stage may suggest differences in differentiation preference toward cTECs or mTECs. Regarding thymic mimetic cells, Huisman and colleagues recently performed high-resolution profiling of human and zebrafish thymic mimetic cells, uncovering both species-specific and species-conserved mimetic cells in human, mouse, and zebrafish thymuses (36). Notably, multiple clusters of muscle and neuroendocrine mimetic cells were found in the human thymus, whereas only a single cluster of each was detected in the mouse thymus, and a single muscle mimetic cluster was noted in the zebrafish thymus (36). Given that autoantibodies in a substantial proportion of MG patients target acetylcholine receptors (AChRs) at neuromuscular junctions, Huisman and colleagues further focused on muscle mimetic cells and found that these mimetic clusters show a differentiation trajectory with a gene signature similar, but not identical, to that of peripheral muscle cells (36). The development of muscle mimetic cells may be regulated by mechanisms similar to those in the periphery, as mouse thymuses deficient in myogenin or Myf5, both of which are myogenic regulatory factors, exhibit an absence or a reduced number of thymic myoid cells (TMCs) (37). Histological analysis of human thymus tissues has shown that some muscle mimetic cells form neuromuscular junction-like structures. Thymic tuft cells expressing choline acetyltransferase (ChAT), an enzyme that synthesizes acetylcholine, are in close proximity to muscle mimetic cells, with their interfaces interspersed with α-bungarotoxin, which binds to AChR (36). However, the significance of the presence of multiple muscle and neuroendocrine mimetic clusters, as well as the formation of neuromuscular junction-like structures in the human thymus, in regulating the immune system, including the establishment of self-tolerance, remains unclear. Furthermore, differences in TEC clusters between human and animal models, such as the number of specific mimetic clusters, may highlight the limitations of translating findings from mouse to human.

The transcriptomic analysis of MG-associated thymomas has led to the identification of a unique mTEC cluster, termed neuromuscular mTECs (nmTECs), which ectopically express neuromuscular molecules and exhibit a transcriptome profile consistent with active antigen presentation (38). Cell–cell interaction analysis has indicated that nmTECs act as a central hub for communication with various cells, including B cells and T cells, via the CXCL12–CXCR4 chemokine axis, as well as non-TEC stroma cells, through such growth factors as vascular endothelial growth factor (VEGF) and platelet-derived growth factor (PDGF) (38). These findings suggest a possible pathogenic role of nmTECs in thymoma-associated MG (TAMG). It is noteworthy that no developmental trajectory from mTECs to “mature myoid cells”—which express desmin and AChRs but not HLA-DR proteins (39)—has been demonstrated by transcriptomic analysis either in thymoma or in the human thymus, although immunohistochemical findings have suggested that such a link is likely in the thymus (8, 39).

## Myasthenia gravis and thymus

MG is an autoimmune disease caused by autoantibodies against components of neuromuscular junctions. MG patients exhibit muscle weakness and fatigability due to impaired neuromuscular transmission (40, 41). Antibodies (Abs) against AChRs are found in 85% of patients (40, 42). In the remaining AChR Ab-negative patients, Abs against muscle-specific kinase (MuSK) are detected in 5% (42, 43), and Abs against lipoprotein receptor-related protein 4 (LRP4) have been reported in a minority of MG patients (44, 45). Another small fraction of patients do not have detectable circulating autoantibodies against known targets. Accordingly, these patients are diagnosed as having seronegative MG.

MG can be clinically divided into the following subtypes on the basis of age of onset, Ab specificity, and associated thymus pathology (**Table 1**): early-onset MG (EOMG); thymoma-associated MG (TAMG); and late-onset MG (LOMG). Seventy percent of EOMG patients exhibit morphological changes in their thymus, particularly thymic hyperplasia (46). Thymoma is observed over a wide age range (47). The thymus in LOMG patients is characterized by age-related involution without histologically recognizable pathology (47). In MuSK Ab-positive MG patients, thymic abnormalities are rare (48, 49). In LRP4 Ab-positive MG patients, thymus involvement remains unclear (50).

**Table 1:** Features and risk factors of myasthenia gravis (MG) subtypes with acetylcholine receptor (AChR) antibodies, comprising early-onset MG (EOMG), thymoma-associated MG (TAMG), and late-onset MG (LOMG).

MG subtype	Autoantigen target	Onset age (years)	Gender bias	HLA association	Thymic pathology	AIRE	Myoid cells
EOMG	AChR	<50	Female-dominant	DR3-B8	TFH	Normal^2^	Normal number
TAMG	AChR, titin Cytokines^1^, RYR1/2	Any (median age approx. 50)	None	None	Thymoma	Reduced^3^	Absent
LOMG	AChR, titin Cytokines^1^, RYR1/2	≥50	Male-dominant	No consistent association	Atrophy	NA	Reduced number

NA, not available; RYR1/2, ryanodine receptors 1 and 2; TFH, thymic follicular hyperplasia.

^1^Cytokines (type I interferons; IL-12).

^2^Supported by the following references (Marx A, et al. Autoimmun Rev (2013) doi: 10.1016/j.autrev.2013.03.007; Scarpino S, et al. Clin Exp Immunol (2007) doi: 10.1111/j.1365-2249.2007.03442.x.; Iacomino N, et al. Biomedicines (2023) doi: 10.3390/biomedicines11030732).

^3^Supported by the following references (Strobel P, et al. J Pathol (2007) doi: 10.1002/patho.2141; Scarpino S, et al. Clin Exp Immunol (2007) doi: 10.1111/j.1365-2249.2007.03442.x.; Iacomino N, et al. Biomedicines (2023) doi: 10.3390/biomedicines11030732).

## EOMG thymus

**Figure 1:**
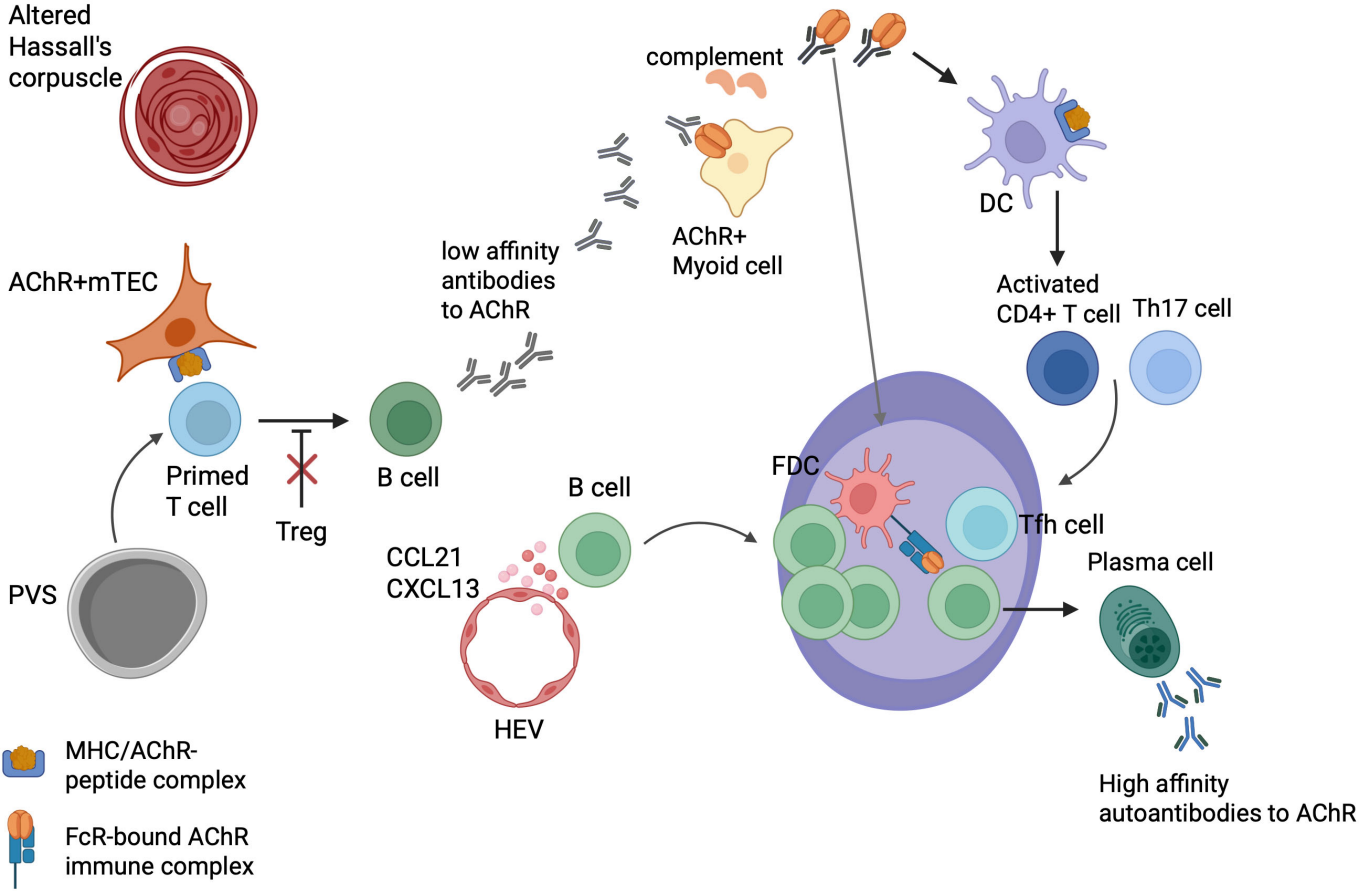
Intrathymic pathogenetic model of early-onset myasthenia gravis. Acetylcholine receptor (AChR)-reactive T cells in blood re-enter the thymus where they, activated by unknown triggers, are “primed” by medullary thymic epithelial cells (mTECs) expressing MHC/AChR–peptide complexes. The primed T cells activate thymic B cells to produce low-affinity anti-AChR Abs. These autoantibodies bind to TMCs expressing native AChRs, activate complement, and induce the release of AChR/Ab complexes from TMCs for processing by nearby dendritic cells (DCs) that bind to follicular dendritic cells (FDCs). CCL21 and CXCL13 facilitate the recruitment of B cells and the formation of ectopic germinal centers (GCs). T follicular helper (Tfh) cells promote GC development, and the GC reaction finally results in plasma cells producing high-affinity anti-AChR Abs. Functionally impaired regulatory T cells (Tregs) may contribute to the maintenance of EOMG. It is unknown whether lymphoid follicles arise primarily in the perivascular space (PVS) or the medulla, and why AChR-reactive T cells occur so commonly in the “physiological” T cell repertoire of healthy humans. This figure is a modification of Figure 3 from Thymus and Autoimmunity, Marx A, Yamada Y, Simon-Keller K, Willcox N, Ströbel P, Weis CA, Semin Immunopathol 43; 45-64 (2021), licensed under a Creative Commons Attribution 4.0 International License (https://creativecommons.org/licenses/by/4.0/).

EOMG may be triggered by genetic predisposition, including HLA genes, miRNA dysregulation, female gender, and immune dysregulation (46, 51–54). The hypothesis that viral infection is a contributor to MG has been posited for a long time, but there is little evidence to support it (42, 55–58). The representative pathogenic finding in EOMG thymus is thymic follicular hyperplasia (TFH), characterized by ectopic lymphoid follicles and germinal centers (GCs) in the perivascular space (PVS) margining with the thymic medulla (59, 60) (**Figure 1**). The exact mechanisms in the early stage of GCs are unknown. However, once formed, GCs drive the hypermutation of B cell receptor genes and the intrathymic production of high-affinity anti-AChR Abs (61). Females exhibit a higher number of GCs, particularly between ages 30 and 40, but the number of GCs decreases after the age of 50, regardless of gender (62). Corticosteroid treatment results in a reduction in the number and size of GCs (63).

### Cellular changes and autoimmunity in EOMG

The medullary area is thought to be a site of immune activation in the MG thymus, where relevant cells, including anti-AChR auto-reactive lymphocytes, TMCs, dendritic cells (DCs), and mTECs, are localized (60). The role of T cells in MG has been demonstrated in previous studies (64–67). B cells develop an anti-AChR autoimmune response upon encountering antigens, interacting with helper T cells (63). Myoid cells are rare non-innervated mesenchymal cells resembling myoblasts or myotubes (68). Myoid cells that are abundant in normal and EOMG thymic medullas express whole native adult and fetal AChRs, but not major histocompatibility complex (MHC) class II molecules (39, 69, 70). TMCs are attacked by active complement and autoantibodies in the EOMG thymus (71, 72). DCs are found in close proximity to myoid cells and likely “cross-present” processed AChR peptides to nearby auto-reactive T cells (73). Hyperplastic mTECs that express unfolded AChR subunits and MHC class II molecules are under attack by complement, and likely prime T cells for immunogenic AChR peptides (74–77). Intrathymic proinflammatory cytokines, CXCL12, CXCL13, CCL21, and B cell-activating factor (BAFF), are overexpressed by increased numbers of activated lymphatic vessels, high endothelial venules, and TECs, and likely contribute to the recruitment of B cells and DCs to the thymus (42, 63, 76, 78–83). The number of thymic Tregs is not decreased; nevertheless, thymic Treg dysfunction is associated with MG pathogenesis (42, 84–86). T follicular helper (Tfh) cells are crucial for the establishment of GC reactions (87). Preoperative immunosuppressive therapy reduces intrathymic Tfh profiles and is suspected to inhibit ectopic GC development (88).

### Pathogenetic model of EOMG and unresolved issues

The early stages of EOMG pathogenesis are still largely unknown. A popular model suggests a two-step pathogenesis (89). In the first step, auto-reactive T cells are activated by mTECs expressing MHC/AChR–peptide complexes. Subsequently, thymic B cells produce low-affinity Abs toward AChRs. In the second step, early Abs attack nearby TMCs expressing folded AChRs on their surface and activate complement, resulting in the subsequent release of AChR/immune complexes. These AChR/immune complexes activate antigen-presenting cells (APCs), such as DCs, which in turn drive the further activation of auto-reactive CD4^+^ T cells. This leads to the initiation of ectopic follicle and GC formation with affinity maturation of their B cell receptors, and ultimately to the production of high-affinity late AChR Abs (89) (**Figure 1**).

The resulting thymic TFH responses may be self-perpetuating, likely due to dysfunctional Tregs in the EOMG thymus and blood (84, 90). Finally, the autoimmune process initiated in the thymus can spread to the periphery, where, hypothetically, skeletal muscle-derived AChR/Ab complexes in regional lymph nodes and functionally defective Tregs may contribute to the maintenance of EOMG (47, 91, 92).

It is believed that mimetic “muscle mTECs” in the human thymus are involved in acetylcholine-mediated interfaces, mimicking peripheral neuromuscular junctions (36). However, it remains unknown whether TMCs are identical to, if not a part of, muscle mTECs.

HCs represent the terminally differentiated stage of mTECs (93–96). The increased number and morphological changes of HCs in the TFH thymus are similar to those in infantile Down syndrome thymus, which exhibits altered AIRE expression (97–99). Down syndrome is associated with impaired Treg function and an increased risk of developing autoimmunity (100, 101). It is conceivable that the altered differentiation of mTECs, including the increase in the number of HCs, may be associated with impaired TEC/thymocyte crosstalk signals in EOMG. Several studies have reported that AIRE expression in the EOMG thymus is comparable to that in the control thymus (47, 102, 103). However, these studies were performed using thymus tissues rather than isolated mTECs. Moreover, other thymic cells, such as B cells or activated DCs, are also known to express AIRE (14, 104). Therefore, AIRE expression in non-mTEC thymic cells may contribute to the apparently undiminished AIRE expression in the EOMG thymus.

Recent single-cell studies have identified specific helper T cells or macrophages in the EOMG thymus (105–107). However, comprehensive single-cell analyses of human TECs and non-TECs from non-neoplastic MG thymus are needed to better understand the underlying immunopathogenesis of EOMG.

## Thymoma-associated MG

TAMG is a subtype of AChR Ab-positive MG (60). Approximately 10 to 20% of generalized AChR Ab-positive MG patients have thymoma (46, 108). TAMG typically occurs after age 50; however, it has a wide age range, including children (109). Unlike EOMG, TAMG shows neither gender distribution nor strong HLA association (47, 50).

**Figure 2:**
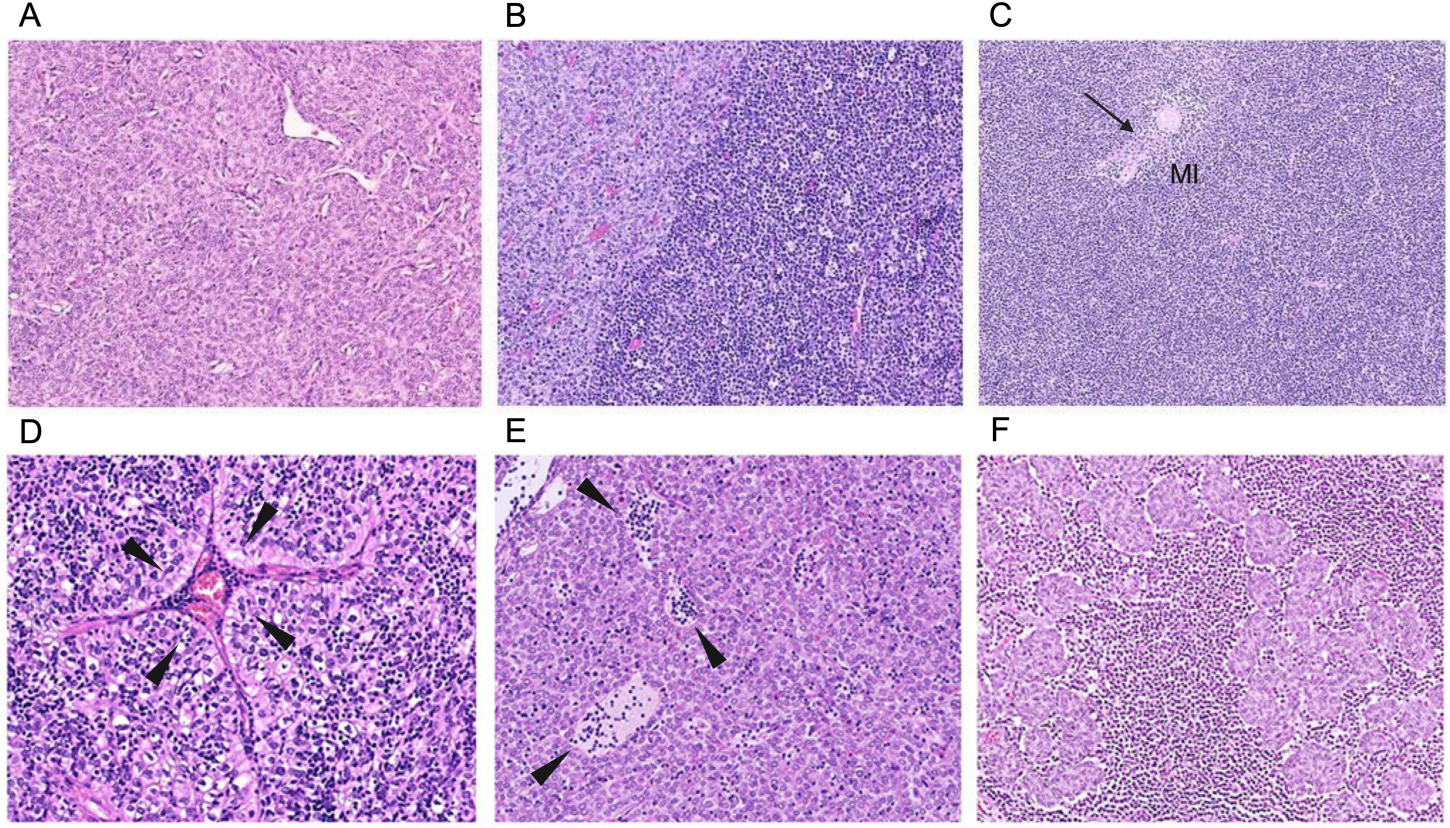
Representative images of typical thymomas (Types A, AB, B1–B3) commonly associated with MG. By definition, types AB, B1, and B2 thymomas consistently harbor interepithelial immature (TdT-positive) T cells, whereas immature T cells are rare and sometimes even absent in types A and B3 thymomas. Enigmatically, the scientifically largely neglected micronodular thymomas with lymphoid stroma are virtually never associated with MG, offering an “experiment of nature” to study the mechanisms driving or preventing the development of thymoma-associated MG. **(A)** Type A thymoma: predominant neoplastic spindle cells and rare (and sometimes no) immature T cells. **(B)** Type AB: biphasic architecture with lymphocyte-poor (left, light) and immature T cell-rich (right, dark) regions. Rarely, lymphocyte-poor regions can be missing altogether. **(C)** Type B1: predominant cortical areas with inconspicuous tumor cells, abundant lymphocytes, and a small medullary island (MI) with a single Hassall corpuscle (HC) (arrow). **(D)** Type B2: many lymphocytes and many conspicuous, large, polygonal epithelial cells arranged in lobules and around a prominent perivascular space (PVS) containing lymphocytes and a blood-filled vessel (arrowheads). **(E)** Type B3: confluent sheets of pink, polygonal tumor cells, few (and eventually no) interepithelial lymphocytes, PVS containing lymphocytes (arrowheads). **(F)** Micronodular thymoma with lymphoid stroma: small nodules of neoplastic epithelial cells resembling those of type A thymoma, surrounded by a lymphoid stroma composed of B cells (often forming follicles) as well as mature and immature T cells.

Thymomas are neoplasms of TECs, characterized by diverse cortical and medullary differentiation accompanied by thymopoiesis in more than 90% of patients (60). The current WHO classification categorizes thymomas into five main types: A, AB, B1, B2, and B3, on the basis of epithelial cell features and lymphocyte content (109). Thymomas containing spindled neoplastic epithelial cells at least focally are classified as type A if they have no or few immature thymic T cells, and as type AB if immature T cells among epithelial cells are at least focally abundant. By contrast, thymomas composed of polygonal tumor cells are classified as type B, and their further division into B1, B2, and B3 depends on the presence of a very high, intermediate, and low number of interepithelial immature T cells, respectively. Types AB, B1, and B2 thymomas are the most prevalent subtypes in MG patients (50) (**Figure 2**). Unlike in EOMG, more than 80% of patients with thymomas have autoantibodies against striational antigens, such as titin and ryanodine receptors (RYRs), as well as others that neutralize cytokines, including type I interferon (IFN) and IL-12 (110, 111). TAMG is the most common thymoma-associated autoimmune disease (30–40%). Others, such as thyroiditis, rheumatoid arthritis, systemic lupus erythematosus, pure red cell aplasia, hypogammaglobulinemia, and other bone marrow failures, are less common (each 1–5%). Together with TAMG, they comprise over 50% of thymoma-associated autoimmune diseases (112, 113).

### Cellular changes and autoimmunity in TAMG

In TAMG, the most common MG-associated thymoma types (AB, B1 to B3) typically show prominent cortex-like regions, with medulla-like regions usually attenuated (114) (**Figure 2**). These thymomas mostly contain abundant immature thymocytes and export high numbers of mature T cells into the peripheral blood, where the export of mature emigrant CD4^+^ T cells is strongly associated with the development of MG (115–117).

Abnormalities in TECs, including the reduced expression of antigen-processing proteases in cTECs and MHC class II antigens in mTECs, as well as defective FOXP3^+^ Treg generation, may contribute to TAMG development (77, 114, 118–120). The loss or reduction of AIRE^+^ mTECs has also been observed in the TAMG thymus (103, 121) (**Table 1**). Molecular components essential for thymic tolerance are deficient in MG thymoma. This may account for the frequent presence of autoantibodies against non-AChR skeletal muscle antigens, including titin and RyR, and against type I IFN (122, 123). The lack of TMCs in thymoma and/or the expression of AChR, titin, and RyR epitopes in neoplastic TECs may also account for the generation of muscle Abs (112, 124, 125). These findings suggest that thymic T cell selection may be altered, or that auto-reactive T cells may be inappropriately activated in the thymic environment.

### Pathogenetic model of TAMG and unresolved issues

The above findings suggest the following pathogenetic model: First, the reduced levels of some HLA variants and neoplastic linear AChR/titin peptide-overexpressing TECs may contribute to altered positive selection (77, 121, 124). Next, auto-reactive thymocytes survive, partly because of the absence of AIRE^+^ mTECs and Tregs, and also because of the combined defects of medullary functions, including a lack of myoid cell-derived AChRs and titin for tolerogenic cross-presentation by APCs (89). Finally, thymoma-derived auto-reactive mature thymocytes escape negative selection in the thymoma, exit into the blood, gradually diluting and eventually replacing the existing tolerant peripheral T cell repertoire (115, 117, 126). In the periphery, including the remnant thymus, these escaping auto-reactive thymocytes stimulate B cells to generate autoantibodies against naïve AChRs after appropriate stimulation. Once initiated, skeletal muscle-derived AChR/autoantibody complexes present in regional lymph nodes perpetuate TAMG even after thymoma removal (108, 124, 127, 128).

Those abnormalities in TECs, which are related to positive and negative selections, are not specific to MG but are commonly detected in thymoma. Despite the loss of AIRE^+^ mTECs, MG is not a common manifestation of human autoimmune polyendocrinopathy–candidiasis–ectodermal dystrophy, which results from various mutations in AIRE (129, 130). Thus, the loss of AIRE^+^ mTECs may be partially, but not entirely, linked to MG pathogenesis. A single-cell sequencing study suggested that a subset of mTECs, named nmTECs, exhibits a significant function through the ectopic expression of neuromuscular molecules in MG thymoma (38). However, nmTEC marker-positive cells are also present in some non-MG thymomas (38). Therefore, the accumulation of neuromuscular-related antigens in nmTECs is not a sufficient condition for MG pathogenesis, and an increased number of nmTECs alone is insufficient to initiate TAMG. Spatial transcriptomic analysis has revealed specific immune niches in the medulla and nmTEC enrichment in the corticomedullary junction (131). Furthermore, a specific chemokine pattern, i.e., CXCL12–CXCR4, and immune cells, including CXCL13^+^ Tfh cells and migratory DCs, have been detected in the MG–thymoma niche (38, 131). Those immune microenvironments, such as CXCL13 interactions, are often observed in TFH (63, 78). Because occasional GCs are enriched by high endothelial venules in TAMG (50, 132), further investigation is needed to elucidate the pathogenesis of TAMG.

## LOMG

There is no consensus on the age threshold for distinguishing between LOMG and EOMG. The most common age threshold of 50 years shows a gender bias distinct from EOMG, i.e., a predominance of males, and a higher frequency of AChR seropositivity (42, 50, 133, 134). LOMG patients, by definition, do not have thymoma. The aging thymus is gradually replaced by fat, but residual parenchyma may continue to export some T cells at least into middle age (135). In LOMG, these remnants may rarely show signs of expansion and even infiltration. However, morphometric analysis did not reveal significant differences between LOMG and normal thymuses (136). TMCs and AIRE-positive cells decline with age. However, there is no apparent difference between LOMG thymuses and age-matched controls (68, 72, 73). Although the genetic background is likely different from that of EOMG and is of pathogenetic relevance, the aged thymus in LOMG is assumed to export and possibly activate non-tolerant T cells (89, 108). Genome-wide association studies (GWAS) have demonstrated that several genes are associated with T cell tolerance (42, 137–139). The following factors are believed to contribute to the pathogenesis of LOMG: 1) immune system aging, which is associated with increased rates of autoimmunity; 2) AChR-reactive T cells, generated in the near absence of myoid cells within a largely AIRE-negative atrophic thymus, may become activated after export to the periphery and subsequently trigger LOMG; and 3) a pathogenic T cell population, derived from an atrophic thymus lacking myoid cells and AIRE expression, is accumulated in the periphery over a long period before the outbreak of LOMG, similar to rare thymoma patients who develop TAMG years after thymoma removal (42, 115). Once initiated, LOMG may become self-perpetuating as described above for TAMG, owing to stimulatory AChR/autoantibody complexes in muscle-draining lymph nodes.

## Other MG subtypes

Patients with MuSK Abs demonstrate a propensity for bulbar muscle involvement (42). Thymoma has been reported as a rare exception in MuSK Ab-positive MG patients (49). Most autoantibodies in MuSK Ab-positive MG are of the IgG4 subclass, in contrast to AChR Abs. Immunopathology of MuSK Abs is less currently known, but the proposed mechanism underlying autoantibody production in MuSK MG is as follows. Peripheral naïve B cells likely encounter self-antigens and receive T cell help in lymphoid tissue. These naïve B cells differentiate into memory B cells and short-lived plasmablasts that produce MuSK Abs (140). LRP4 Abs are primarily of IgG1 and IgG2 subtypes and are associated with clinical presentations resembling the mild form of EOMG (141). Patients without these other autoantibodies, namely triple seronegative MG represent a highly heterogeneous group, and there is limited information regarding their disease mechanisms (42).

## Thymectomy

Thymectomy is a standard treatment option in AChR Ab-positive MG. It should be performed as early as possible, ideally within two years of MG onset (142, 143). Thymectomy can effectively remove AChR-like proteins, antigen-specific T cells, and Ab-producing B cells (144–146). Whereas clinical improvement is observed in half of patients following thymectomy, complete remission is rare (147, 148). Potent autoantibody-producing B cells can differentiate into long-lived plasma cells in the thymus, leading to the production of some of the circulating AChR-specific autoantibodies (140, 149, 150). Thymus-derived B cell clones persist in the circulation after thymectomy, and these B cells are thought to be associated with poor outcome (151). Thymectomy has not resulted in clinical improvement in MuSK Ab-positive patients, unlike in AChR Ab-positive patients (152, 153). MuSK Ab-positive thymus shows few GCs, and these thymic changes are thought to be associated with responses to thymectomy (48). Several studies have demonstrated that rituximab, which depletes B cells, achieves a higher improvement rate in MuSK Ab-positive MG than in AChR Ab-positive MG. However, rituximab has been reported to reduce the risk of relapse in AChR Ab-positive MG, although its benefit appears greater in MuSK Ab-positive MG (154). It is speculated that autoantibody-producing B cell clones residing in AChR Ab-positive MG thymus can also populate lymphoid tissues outside the thymus. In those cases, there may be pathogeneses in which thymectomy or rituximab is ineffective.

## Conclusions and perspectives

Thymic abnormalities are observed in a substantial proportion of patients with MG, prompting extensive investigation into the involvement of the thymus in the immunopathogenesis of the disease. Recent studies have shown the expanding diversity of TEC subpopulations in relation to neuromuscular junctions. ChAT-expressing thymic tuft cells are in close proximity to muscle mimetic cells in the normal human thymus. However, the role of these TEC subpopulations in the MG thymus remains to be investigated. Further studies are required to determine whether muscle mTECs are identical to, or represent a subset of, TMCs, and whether a specific TEC subpopulation, such as muscle mTECs, expresses immunogenic AChRs in the thymus of MG patients.

The understanding of TEC biology in humans lags behind that in mouse models. In this regard, the development of methods for purifying TEC subpopulations from both non-thymomatous and neoplastic thymic tissues is highly anticipated, as this will facilitate the identification of triggers leading to MG. Experimental autoimmune MG (EAMG) animal models have been established to investigate pathogenic mechanisms (155). However, these EAMG animal models lack thymic abnormalities, limiting their utility in elucidating the pathogenic mechanisms associated with the abnormal thymus in MG. A comprehensive understanding of TEC biology in the MG thymus will positively impact the engineering of EAMG animal models with thymic abnormalities, thereby further advancing research on MG thymus.

Thymectomy is one of the long-acting immunotherapies. However, it is currently not possible to predict the postoperative MG course owing to the lack of preoperative biomarkers and predictive morphological features of the resected thymic tissue. The problem with performing scRNA-seq/spatial transcriptomic analysis of MG thymus lies in the preoperative use of corticosteroids, which can lead to pathological changes in the thymus. However, the integration of recent advancements, such as *in situ* single-nucleus barcoding, may provide a solution to this problem. Biomarkers should aim at identifying highly active and refractory MG patients. Given the expanding treatment landscape in MG, highly disease-active and refractory cases should be considered for new targeted therapies, such as complement inhibitors or B cell depletion. We hope that thymus research will contribute to a better understanding of MG pathogenesis and enable the establishment of biomarkers for MG-specific therapy that interrupt AChR/MuSK-directed autoimmunity without compromising other aspects of immune function.
